# Thiosemicarbazone-Based Compounds: A Promising Scaffold for Developing Antibacterial, Antioxidant, and Anticancer Therapeutics

**DOI:** 10.3390/molecules30010129

**Published:** 2024-12-31

**Authors:** Agnieszka Czylkowska, Monika Pitucha, Anita Raducka, Ewelina Fornal, Edyta Kordialik-Bogacka, Sylwia Ścieszka, Marek Smoluch, Franciszek Burdan, Mateusz Jędrzejec, Paweł Szymański

**Affiliations:** 1 Institute of General and Ecological Chemistry, Faculty of Chemistry, Lodz University of Technology, Żeromskiego 116, 90-924 Lodz, Poland; anita.raducka@p.lodz.pl (A.R.); ewelina.fornal@dokt.p.lodz.pl (E.F.); 2 Independent Radiopharmacy Unit, Faculty of Pharmacy, Medical University of Lublin, Chodzki 4a, 20-093 Lublin, Poland; monika.pitucha@umlub.pl; 3 Institute of Fermentation Technology and Microbiology, Faculty of Biotechnology and Food Sciences, Lodz University of Technology, Wólczańska 171/173, 90-924 Lodz, Poland; edyta.kordialik-bogacka@p.lodz.pl (E.K.-B.); sylwia.scieszka@p.lodz.pl (S.Ś.); 4 Faculty of Materials Science and Ceramics, AGH University, Mickiewicza 30, 30-059 Krakow, Poland; smoluch@agh.edu.pl; 5 Human Anatomy Department, Medical University of Lublin, Jaczewskiego 4, 20-090 Lublin, Poland; franciszek.burdan@umlub.pl; 6 Department of Pharmaceutical Chemistry, Drug Analyses and Radiopharmacy, Faculty of Pharmacy, Medical University of Lodz, 90-151 Lodz, Poland; mateusz.jedrzejec@umed.lodz.pl (M.J.); pawel.szymanski@umed.lodz.pl (P.S.); 7 Department of Radiobiology and Radiation Protection, Military Institute of Hygiene and Epidemiology, 01-163 Warsaw, Poland

**Keywords:** thiosemicarbazone, antibacterial activity, antioxidant, anticancer therapeutics

## Abstract

This paper presents the synthesis and characterization of new thiosemicarbazone derivatives with potential applications as antibacterial, antioxidant and anticancer agents. Six thiosemicarbazone derivatives (L–L5) were synthesized by reacting an appropriate thiosemicarbazide derivative with 2-pyridinecarboxaldehyde. The structures of the obtained compounds were confirmed using mass spectrometry, infrared spectroscopy, and NMR spectroscopy. Antibacterial activity was evaluated by using the microdilution method, determining the minimum inhibitory concentration (MIC) against a panel of Gram-positive and Gram-negative bacteria. Compound L1 showed the most potent antibacterial activity, especially against *Bacillus cereus* (MIC 10 mg/L). Molecular docking to topoisomerase II and transcriptional regulator PrfA suggests that the studied compounds can effectively bind to molecular targets recognized in anticancer and antibacterial therapies. An assessment of physicochemical properties (ADME) indicates favorable parameters of the compounds as potential drugs. Compounds L and L2 showed the highest antioxidant activity, surpassing the activity of the Trolox standard. Cytotoxicity against A549 lung cancer cells was evaluated by the MTT assay. Compound L4 exhibited the strongest inhibitory effect on cancer cell survival. The obtained results indicate that the synthesized thiosemicarbazide derivatives, especially L1, L2, and L4, are promising compounds with potential applications as antibacterial and anticancer drugs.

## 1. Introduction

Thiosemicarbazones are chemical compounds that have been of interest to scientists since the 19th century [1] due to their diverse biological properties, which play a significant role in organic chemistry and medicine. These compounds contain a thiosemicarbazide group (-NH-CS-NH-NH2) that is crucial for their biological activities, such as antibacterial [2,3], antitumor [4,5,6], antioxidant [7,8], antifungal [9,10], antiviral [11,12], and anti-inflammatory behavior [13,14]. In recent years, thiosemicarbazones have gained considerable attention from scientists due to their potential applications in the treatment of infectious and neoplastic diseases [15,16]. Both thiosemicarbazones and thiosemicarbazides exhibit antibacterial properties, making them promising candidates for developing new antibiotics [17,18,19]. Molecular studies and docking simulations suggest that thiosemicarbazides may act through a dual mechanism involving the inhibition of DNA gyrase and topoisomerase IV [20,21], which leads to the disruption of DNA replication and bacterial cell death. Thiosemicarbazides also have anticancer properties [22,23,24]. It has been shown that the mechanism of their anticancer action involves the induction of apoptosis (programmed cell death) [25]. Studies on thiosemicarbazones have shown that some of these compounds exhibit significant anticancer activity against various cell lines. For example, thiosemicarbazones were tested on lung cancer (A549) [26,27,28] and cervical cancer (HeLa) cell lines, where they demonstrated the ability to inhibit cancer cell proliferation and induce apoptosis. These studies make them promising candidates for further research and development of new anticancer therapies. Furthermore, thiosemicarbazones have antifungal properties [29,30,31]. The mechanism of their antifungal action involves disrupting the function of fungal cell membranes and inhibiting protein synthesis, leading to the death of fungal cells. Studies on various compounds have demonstrated their effectiveness in combating various fungal pathogens, such as *Aspergillus flavus*, suggesting their potential application in treating fungal infections [32]. In terms of chemical modifiability, thiosemicarbazones have an advantage, as they can be easily modified to improve their properties and synthesize new compounds with potential therapeutic applications [19,33]. In contrast, natural compounds have limited possibilities for modification which require complex biosynthetic pathways [34]. Regarding stability and consistency, thiosemicarbazones are more predictable due to synthetic processes ensuring uniformity [35,36,37]. Although the toxicity of thiosemicarbazones can be unexpected, it is crucial to understand that it may vary depending on the application and chemical structure. Isatin-thiosemicarbazone derivatives have shown promising results in reducing neurotoxicity in Alzheimer’s disease models, showing low toxicity at effective therapeutic concentrations [38]. Acridine-thiosemicarbazone derivatives have been studied for anticancer activity, showing significant cytotoxicity against cancer cells but low acute toxicity in nonclinical models, indicating their potential as chemotherapeutic agents [39]. Thus, thiosemicarbazones may exhibit toxicity depending on structure and application, but many of them have a favorable safety profile, making them promising candidates for further therapeutic development [40].

In our paper, we present the synthesis and characterization of six thiosemicarbazone derivatives. Although the compounds L, L1, and L3 were previously obtained and described [41,42,43], our research fills the gap in the literature regarding these types of compounds.

## 2. Results and Discussion

### 2.1. Synthesis and Physicochemical Characterization

**Figure 1:**
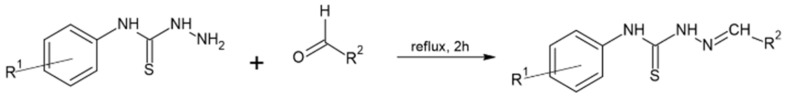
Scheme of the synthesis of compounds **L–L5**.

The ligand was prepared using a modified method described previously [44], in which a solution of an appropriate thiosemicarbazide derivative (1 mmol) in methanol (20 mL), a solution of 2-pyridinecarboxaldehyde (1 mmol), and a few drops of HCl were slowly added. The resulting mixture was heated under reflux for 2 h. The precipitate was then filtered off, washed with methanol, and recrystallized from methanol. The following were used as thiosemicarbazides: 4-phenyl-3-thiosemicarbazide, 4-(2-chlorophenyl)-3-thiosemicarbazide, 4-(3-chlorophenyl)-3-thiosemicarbazide, 4-(4-chlorophenyl)-3-thiosemicarbazide, 4-(2,4-dichlorophenyl)-3-thiosemicarbazide, and 4-(2,6-dichlorophenyl)-3-thiosemicarbazide. 2-Pyridinecarboxaldehyde was used as a carbonyl compound (Figure 1, Table 1). The synthesis of these compounds has been optimized compared to those described in the literature [41,42,43].

**Figure 2:**
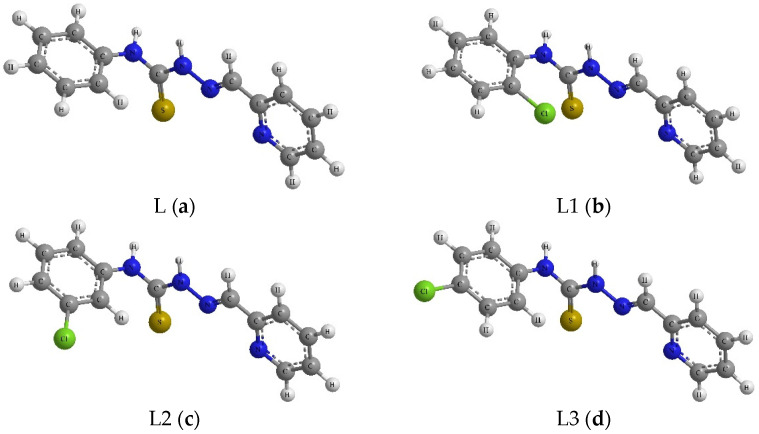

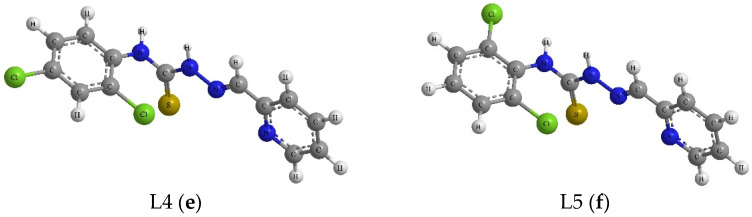
The structural formula of compounds **L–L5**. (**a**) The structural formula of compound L: (N-phenyl-2-(pyridin-2-ylmethylene)hydrazine-1-carbothioamide). (**b**) Structural formula of compound L1: (N-(2-chlorophenyl)-2-(pyridin-2-ylmethylene)hydrazine-1-carbothioamide). (**c**) Structural formula of compound L2: (N-(3-chlorophenyl)-2-(pyridin-2-ylmethylene)hydrazine-1-carbothioamide). (**d**) Structural formula of compound L3: (N-(4-chlorophenyl)-2-(pyridin-2-ylmethylene)hydrazine-1-carbothioamide). (**e**) Structural formula of compound L4: (N-(2,4-dichlorophenyl)-2-(pyridin-2-ylmethylene)hydrazine-1-carbothioamide). (**f**) Structural formula of compound L5: (N-(2,6-dichlorophenyl)-2-(pyridin-2-ylmethylene)hydrazine-1-carbothioamide).

The structural formulas of the obtained compounds are presented in Figure 2a–f. All six compounds were thoroughly characterized using appropriate analytical techniques, including ^1^H and ^13^C NMR spectroscopies (Figure S1a–l), mass spectrometry (Figure S2a–f), and FTIR spectroscopy (Figure S3a–f).

Due to the structural similarities, the obtained spectra and fragmentation patterns display visible resemblances. The obtained results confirm the high purity of the synthesized compounds. Table 2 lists high-resolution mass spectrometry results compared to the theoretical values.

L: C_13_H_12_N_4_S (256.326 g/mol), yield: 89%, m.p. 184–186 °C. ^1^H NMR (DMSO-d_6_): 7.19–7.66 (m, 5H, CH_phenyl_), 7.84–7.86 (m, 1H, CH_pyridine_), 8.11 (s, 1H, CH), 8.45–8.47 (m, 1H, CH_pyridine_), 8.59–8.79 (m, 2H, CH_pyridine_), 10.27 (s, 1H, NH), 12.05 (s, 1H, NH). ^13^C NMR: 121, 125, 126, 127, 128, 136, 134, 138, 139, 143, 149, 153, 176 [34]. ESI-MS *m*/*z*: [M+H]^+^ theoretical: 257.0855, measured: 257.0847. FTIR (cm^−1^): v(NH): 3308; v(CC): 1600, 1553; v(CN), δ(CH): 1467, 1434, 1326, 1257, 1189, 998, 754, 693.

L1: C_13_H_11_N_4_SCl (290.771 g/mol), yield: 88%, m.p. 173–176 °C. ^1^H NMR (DMSO-d_6_): 7.32–7.65 (m, 4H, CH_phenyl_), 7.84–7.85 (m, 1H, CH_pyridine_), 7.86–7.87 (s, 1H, CH_pyridine_), 8.21 (s, 1H, CH), 8.38–8.39 (m, 1H, CH_pyridine_), 8.59–8.60 (m, 1H, CH_pyridine_), 10.24 (s, 1H, NH), 12.20 (s, 1H, NH). ^13^C NMR: 120, 124, 125, 127, 128, 129, 130, 134, 136, 143, 148, 152, 177 [35]. ESI-MS *m*/*z*: [M+H]^+^ theoretical: 291.0466, measured: 291.0458. FTIR (cm^−1^): v(NH): 3268; v(CC): 1591, 1538; v(CN), δ(CH): 1468, 1435, 1322, 1294, 1148, 1186, 1034, 998; v(CN), δ(CH), v(CCl): 754.

L2: C_13_H_11_N_4_SCl (290.771 g/mol), yield: 89%, m.p. 162–164 °C. ^1^H NMR (DMSO-d_6_): 7.25–7.75 (m, 4H, CH_phenyl_), 7.84–7.88 (m, 2H, CH_pyridine_), 8.11 (s, 1H, CH), 8.22–8.45 (m, 1H, CH_pyridine_), 8.59–8.80 (m, 1H, CH_pyridine_), 10.32 (s, 1H, NH), 12.18 (s, 1H, NH). ^13^C NMR: 121, 124, 125, 127, 129, 130, 132, 134,140, 144, 149, 152, 176. ESI-MS *m*/*z*: [M+H]^+^ theoretical: 291.0466, measured: 291.0458. FTIR (cm^−1^): v(NH): 3316; v(CC): 1585, 1543; v(CN), δ(CH): 1482, 1439, 1390, 1251, 1194, 1076, 998, 854; v(CN), δ(CH), v(CCl): 779.

L3: C_13_H_11_N_4_SCl (290.771 g/mol), yield: 77%, m.p. 194–196 °C. ^1^H NMR (DMSO-d_6_): 7.33–7.62 (m, 4H, CH_phenyl_), 7.81–7.87 (m, 1H, CH_pyridine_), 8.12 (s, 1H, CH), 8.63–8.68 (m, 2H, CH_pyridine_), 8.70–8.89 (m, 1H, CH_pyridine_), 10.27 (s, 1H, NH), 12.49 (s, 1H, NH). ^13^C NMR: 121, 122, 127, 128, 129, 130, 131, 136, 140, 141, 150, 151, 177 [36]. ESI-MS *m*/*z*: [M+H]^+^ theoretical: 291.0466, measured: 291.0456. FTIR (cm^−1^): v(NH): 3315; v(CC): 1588, 1541; v(CN), δ(CH): 1489, 1468, 1394, 1258, 1192; v(CN), δ(CH), v(CCl): 833, 775; δ(CH), v(CCl): 674.

L4: C_13_H_10_N_4_SCl_2_ (325.261 g/mol), yield: 72%, m.p. 204–206 °C. ^1^H NMR (DMSO-d_6_): 7.39–7.74 (m, 3H, CH_phenyl_), 7.84–7.88 (m, 1H, CH_pyridine_), 8.11 (s, 1H, CH), 8.21–8.40 (m, 2H, CH_pyridine_), 8.59–8.60 (m, 1H, CH_pyridine_), 10.23 (s, 1H, NH), 12.25 (s, 1H, NH). ^13^C NMR: 120, 125, 127, 129, 132, 135, 136, 138, 143, 148, 152, 153, 177. ESI-MS *m*/*z*: [M+H]^+^ theoretical: 325.0076, measured: 325.0065. FTIR (cm^−1^): v(NH): 3272; v(CH): 3061; v(CC): 1578, 1531; v(CN), δ(CH): 1467, 1321, 1192, 857; v(CN), δ(CH), v(CCl): 800; δ(CH), v(CCl): 696.

L5: C_13_H_10_N_4_SCl_2_ (325.261 g/mol), yield: 85%, m.p. 195–198 °C. ^1^H NMR (DMSO-d_6_): 7.36–7.60 (m, 3H, CH_phenyl_), 8.11 (s, 1H, CH), 8.20–8.44 (m, 2H, CH_pyridine_), 8.60–8.80 (m, 2H, CH_pyridine_), 10.27 (s, 1H, NH), 12.24 (s, 1H, NH). ^13^C NMR: 121, 124, 125, 128, 129, 135, 136, 143, 148, 153, 178. ESI-MS *m*/*z*: [M+H]^+^ theoretical: 325.0076, measured: 325.0069. FTIR (cm^−1^): v(NH): 3248; v(CH): 3119, 2968, v(CC): 1537, 1506; v(CN), δ(CH): 1468, 1436, 1270, 1103, 1077, 939; v(CN), δ(CH), v(CCl): 785.

### 2.2. Molecular Docking

**Figure 3:**
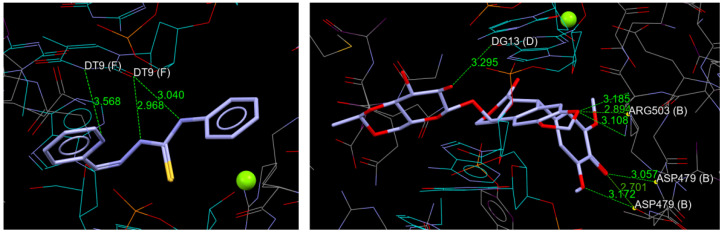
L compound (**left**) and the original inhibitor (EVP, etoposide) (**right**) docked to the active site of TopoII (B chain).

**Figure 4:**
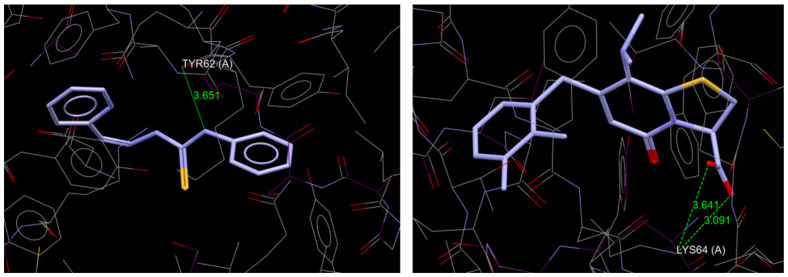
L compound (**left**) and the original inhibitor (QQH) (**right**) docked to the active site of PrfA.

The possible interactions of the analyzed compounds were assessed based on two well-recognized molecular targets in anticancer and antimicrobial studies. Topoisomerases are among the most important targets in anticancer research due to their essential role in the DNA transcription process [45]. The transcriptional regulator PrfA is a crucial target for antibacterial studies due to its pivotal role in controlling virulence gene expression and environmental adaptability [46]. The docking scores are represented as the total ChemPLP model fitness value of a ligand docked to a target molecule. The obtained values (Table 3) suggest that all compounds can effectively bind to the proposed biomolecules. The highest scores were obtained for L-TopoII and L-PrfA complexes. In both cases, the ligand-target structures are stabilized with intermolecular hydrogen bonds. In the case of L-TopoII, these bonds are formed between the thiosemicarbazone NH groups and C=O group in DT9 (F) residue, as well as between the pyridine nitrogen atom and NH group in DT9 (F) residue. For the L-PrfA complex, the hydrogen bond is formed between one of the thiosemicarbazone NH group and the C=O group in Tyr62 (A). The results were visualized in 3D (Figure 3 and Figure 4), along with the original inhibitors docked to the active sites of the appropriate molecular targets.

### 2.3. In Silico Biological Activity Predictions

The compounds need to possess basic features that indicate their potential biological utility. All six compounds were evaluated in silico, and all analyses and conclusions were drawn based on theoretical computational models. This method relies heavily on the Swiss ADME method (Absorption, Distribution, Metabolism, and Excretion) of analysis to predict the behavior of these compounds in biological systems. The findings derived from these computational models provide valuable insights into the potential efficacy and safety of the compounds with potential biological activity.

**Figure 5:**
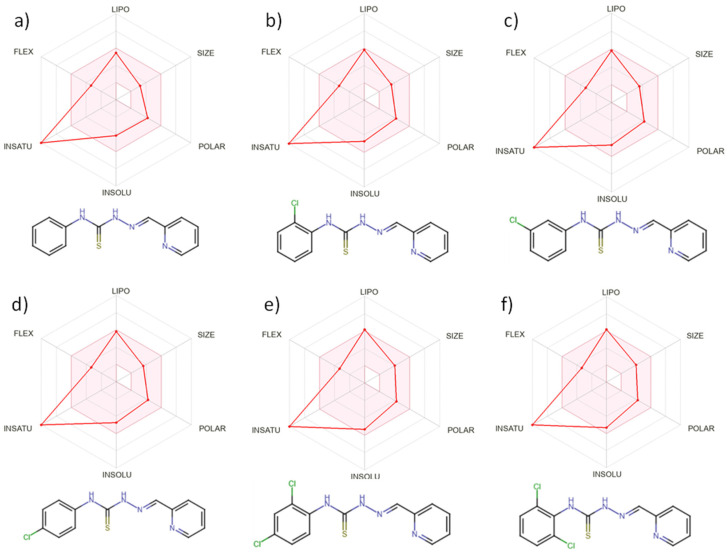
Bioavailability radars for compounds (**a**) L; (**b**) L1; (**c**) L2; (**d**) L3; (**e**) L4; (**f**) L5.

**Figure 6:**
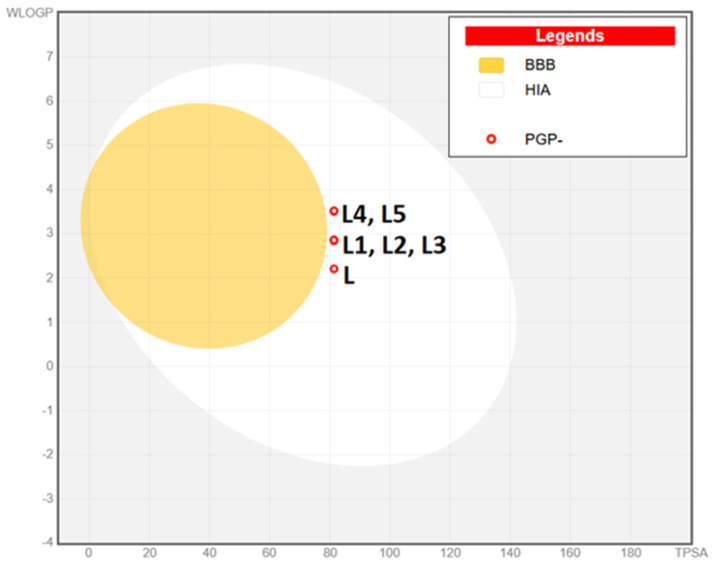
Boiled—egg diagram for the tested compounds. BBB—blood-brain barrier permeation; HIA—high gastrointestinal absorption.

This theoretical framework allows for the rapid screening of numerous compounds, facilitating early-stage drug discovery while minimizing resource expenditure and ethical concerns associated with animal testing. Each studied chemical corresponds to Veber’s [47] and Lipinski’s [48] guidelines. Six physicochemical features are shown by bioavailability radars (Figure 5a–f): lipophilicity, size, polarity, solubility, flexibility, and saturation. High gastrointestinal absorption is predicted for all based on theoretical computational models’ compounds. None of the tested compounds is predicted to permeate the blood-brain barrier (Figure 6). All substances have an acceptable bioavailability score of 0.55, which is determined by considering the Lipinski filter violation, TPSA, and total charge. Each compound is classified as being of the fourth toxicity class.

### 2.4. Antibacterial Activity

The antibacterial activity of the tested samples against the referenced microorganisms varied depending on the chemicals being examined (Table 4). *Salmonella typhimurium* ATCC 14028 and *Enterococcus faecalis* ATCC 29212 were not sensitive to the tested substances, with MIC values exceeding 1000 mg/L. Compound L demonstrated the strongest antibacterial properties against *Bacillus subtilis* ATCC 6633, *Staphylococcus epidermidis* ATCC 12228, and *Listeria monocytogenes* ATCC 19115 (MIC 200 mg/L). The lowest MIC (10 mg/L) was obtained for compound L1 against *Bacillus cereus* ŁOCK 0807. L1 also showed high antibacterial activity against *Staphylococcus aureus* ATCC 6538, *S. epidermidis* ATCC 12228, and *L. monocytogenes* ATCC 19115 (MIC 100 mg/L). Compound L2 exhibited antagonistic activity against *B. subtilis* ATCC 6633 and *S. aureus* ATCC 25923 (MIC 50 mg/L). Compound L3 showed antibacterial activity against *Bacillus* spp. (MIC values of 100 mg/L for *B. cereus* ŁOCK 0807 and 200 mg/L for *B. subtilis* ATCC 6633), while the antagonistic activity of compound L4 was observed against *B. cereus* ŁOCK 0807 (MIC 50 mg/L) and *S. aureus* ATCC 6538 (MIC 100 mg/L). Compound L5 exhibited the lowest antibacterial activity among the tested chemicals. The reference positive controls used in this study were antibiotics: vancomycin for Gram-positive bacteria and ciprofloxacin for Gram-negative bacteria (*E. coli*, *S.* Typhimurium). A 50% (*v*/*v*) DMSO solution was used as a negative control.

### 2.5. Antioxidant Activity

#### 2.5.1. ABTS

Compounds L and L2 have lower IC50 levels than STD. However, the compounds L1, L3, and L5 have the same or similar activity as Trolox (Table 5). These molecules are good antioxidant compounds and candidates for drugs in terms of antioxidant properties. In conclusion, these results show that these two molecules (L and L2) have the best antioxidant properties of all tested molecules [49,50].

#### 2.5.2. DPPH

As we can see, the results show (Table 6) that one compound, L2, has comparable antioxidant properties to Trolox, which was used as Standard (STD). Comparing the IC50 value of L1 and L2 to the IC_50_ of Trolox, we can conclude that both chemical substances have a similar levels of antioxidant properties like Standard. The difference between the IC_50_ values is small, therefore, these molecules can be good antioxidant compound and medicine candidates in terms of antioxidant properties. The result of L3 was rejected because the absorbances for all 9 different concentrations were indeterminate [50,51].

#### 2.5.3. ORAC-FL

The results of this analysis were obtained using the ORAC-FL method. In this method, the kinetics of the oxidation reaction were checked. Trolox was used as an internal standard to calculate Trolox equivalents (TE) [51]. All results were averaged (Table 7). The Oxygen Radical Absorbance Capacity (ORAC) assay is a method that measures the antioxidant capacity of substances, indicating that compound L1 has the highest activity in this respect. However, substances L2, L3, and L4 have comparable activity. Analyzing the basic substance L, it can be concluded that its activity is the weakest and similar to the structural changes leading to the L5 derivative. Therefore, the most favorable results in the ORAC-FL test are structural changes leading to the L1 derivative and then to L2, L3, and L4.

#### 2.5.4. Cell Culture and MTT Cytotoxicity Assay

**Figure 7:**
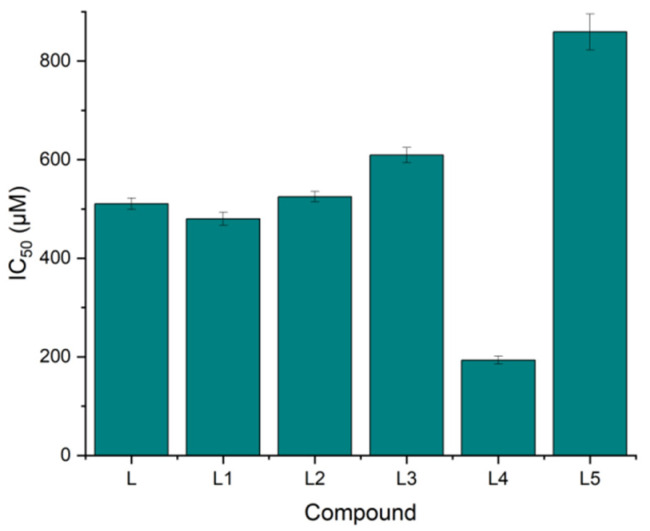
Comparison of cell survival in relation to the ligands and substituents used.

The cytotoxicity test involved checking how changes in substituents and ligands affect cell survival. As a screening analysis, this study was not intended to demonstrate the impact of potential anticancer activity but the relationship between individual derivatives and the ligand. For this reason, small-cell lung cancer cells (A549) were selected as the cell type most studied due to the high incidence of this type of cancer. As a result (Table 8) of the conducted research, it is evident that introducing L1 and L2 substituents does not significantly affect cell survival. Therefore, it can be concluded that the potential impact of the introduced changes to the ligand is negligible, and the safety of use and the potential toxic effect of compounds L, L1, and L2 will be at a comparable level. The introduction of the L4 and L5 substituents had the most significant impact on cell viability, significantly influencing the potential safety of the obtained L5 derivative. It is important that it has been shown that substituents from group L5, when introduced into the basic chemical system, can be derivatives with a higher safety profile. However, the L4 compound is an excellent derivative as an anticancer substance. A comparison of cell survival in relation to the used ligands and substituents is presented in Figure 7.

## 3. Materials and Methods

### 3.1. Chemicals

All chemicals used for the synthesis were purchased from Sigma-Aldrich (St. Louis, MO, USA), Alfa Aesar (Haverhill, MA, USA), and POCH (Gliwice, Poland) companies and used without further purification.

### 3.2. Mass Spectrometry

An Exploris 240 (Thermo Scientific, Bremen, Germany) mass spectrometer equipped with an electrospray ionization source was used. Positive-ion electrospray was performed at a spray voltage of 3.4 kV. Spectral acquisition was performed between *m*/*z* 80 and 800 in full scan mode. Data were analyzed using FreeStyle 1.8 (Thermo Scientific, Waltham, MA, USA) software. Samples were dissolved and diluted in methanol to achieve a 1 µg/mL concentration. The Ultimate 3000 system (Thermo Scientific, Bremen, Germany) was then used to inject 1 µL of each sample at a flow rate of 20 µL/min.

### 3.3. Infrared Spectra

FTIR spectra were recorded with an IR Tracer-100 Schimadzu Spectrometer (4000–600 cm^−1^ with recording accuracy of 1 cm^−1^, Schimadzu, Kyoto, Japan) using KBr pellets.

### 3.4. Minimum Inhibitory Concentration

The antimicrobial activity, as indicated by the minimum inhibitory concentration (MIC) of the synthesized compounds, was evaluated using both the dilution and the diffusion-well method. The tested compounds were dissolved in a 50% (*v*/*v*) DMSO solution, which was recommended due to their chemical structures. Since this solvent can exhibit antimicrobial activity, a control with serial dilutions of DMSO was also included in the tests. The antimicrobial activity of synthesized compounds was only considered after excluding the impact of DMSO. The MICs of the tested samples were evaluated against a panel of reference microorganisms obtained from the American Type Culture Collection (ATCC), including Gram-negative bacteria (*Escherichia coli* ATCC 10530, *Salmonella typhimurium* ATCC 14028), and Gram-positive bacteria (*Staphylococcus aureus* ATCC 25923, *S. aureus* ATCC 6538, *Staphylococcus epidermidis* ATCC 12228, *Enterococcus faecalis* ATCC 29212, *Listeria monocytogenes* ATCC 19115, *Bacillus subtilis* ATCC 6633), as well as one strain (*Bacillus cereus* ŁOCK 0807) obtained from the Pure Culture of Industrial Microorganisms of the Institute of Fermentation Technology and Microbiology ŁOCK 105 (Łódź, Poland). Vancomycin and ciprofloxacin (Sigma-Aldrich, Saint Louis, MO, USA) were used as reference antibiotics (positive controls).

A set of 24 h bacterial cultures with a density of 1.5 × 10^8^ CFU/mL (corresponding to a McFarland standard of 0.5) were plated (0.1 mL) on Mueller–Hinton Agar (Merck, Darmstadt, Germany) following the procedures recommended by the European Committee on Antimicrobial Susceptibility Testing (EUCAST) [52]. Wells with a diameter of 8 mm were cut in the agar medium. Subsequently, 50 µL of prepared solutions containing the tested compounds at concentrations ranging from 10,000 mg/L to 1 mg/L dissolved in a 50% (*v*/*v*) DMSO were added to each well. The plates were incubated at 30 °C (for *B. subtilis* and *B. cereus*) or 37 °C (for other bacteria) for 18 h. After incubation, the MIC value (the lowest concentration of the tested compounds that prevents bacterial growth) was determined and expressed in mg/L. The experiment was conducted in three repetitions.

### 3.5. Molecular Docking

Molecular docking of the studied compounds was performed using GOLD and Hermes 2023.2.0 software [53,54,55]. The crystal structures of the selected target molecules were obtained from the Protein Data Bank: DNA-Topoisomerase IIβ complex (Topo II, PDB ID: 3QX3) and transcriptional regulator PrfA from *L monocytogenes* (PrfA, PDB ID: 6EXM). The ligand molecules were optimized with Gaussian software (http://wild.life.nctu.edu.tw/~jsyu/compchem/g09/g09ur/m_citation.htm accessed on 17 November 2024) using the B3LYP/6-31G method [56]. Before docking, all water molecules and bound ligands were removed, and hydrogen atoms were added. The binding properties were calculated with the ChemPLP scoring function for the area within a radius of 8.0 Å from the original ligand. For method validation, the original ligands were re-docked to the appropriate target (PDB ID: EVP ligand for 3QX3 target; PDB ID: QQH ligand for 6EXM target). For docking simulations, all studied ligands were set as flexible molecules.

### 3.6. ADME

The druglikeness, pharmacokinetic profile, and overall toxicity of L–L5 compounds were evaluated using freely available online tools: the SwissADME service (Swiss Institute of Bioinformatics 2021) [57,58] and the ProTOX II service [59].

### 3.7. ABTS

The antioxidant activity of the selected compounds was measured using a 2,2′-azino-bis(3-ethylbenzothiazoline-6-sulfonic acid)—ABTS (Sigma-Aldrich, Steinheim, Germany). In this research, ABTS (2,2′-azino-bis(3-ethylbenzothiazoline-6-sulfonic acid)) was used. K_2_S_2_O_7_, which activates ABTS from nonactive to an active form, and 6-hydroxy-2,5,7,8-tetramethyl-chromane-2-carboxylic acid (Trolox) as a standard were purchased from Sigma-Aldrich (Steinheim, Germany). The assays were carried out in 75 mM phosphate buffer (pH 7.4) with the final reaction volume of 50 µL. All reagents were first dissolved in dimethyl sulfoxide (DMSO) and then diluted in 75 mM phosphate buffer (pH 7.4). The final concentrations were 0.01–25 µL/mL for Trolox and compounds. A solution of the tested compound/Trolox (50 µL) and ABTS solution in final concentration was placed in the 96-well microplate (Greiner Bio-One. Frickenhausen, Germany). The plate was immediately placed in a multifunctional microplate reader (Synergy H1, BioTek, Santa Clara, CA, USA). Measurements of the absorbance were made at room temperature using a microplate reader. The wavelength was 734 nm. As a blank, 75 mM phosphate buffer (pH 7.4) was used. The ABTS stability during measurements was assessed by the addition of 75 mM phosphate buffer (pH 7.4) instead of the tested compounds/Trolox. The plate was automatically shaken before each measurement for 5 s. All tests were performed in triplicate for each sample [60].

### 3.8. DPPH

The antioxidant activity of the selected compounds was measured using 2,2-diphenyl-1-picrylhydrazyl (DPPH) as an oxidant factor. 6-hydroxy-2,5,7,8-tetramethyl-chromane-2-carboxylic acid (Trolox) was used as a standard. The assays and standard were dissolved in 75 mM phosphate buffer (pH 7.4) with a final reaction volume of 50 µL in each well. All reagents were dissolved firstly in dimethyl sulfoxide (DMSO) and then diluted in 75 mM phosphate buffer (pH 7.4). The final concentrations were 0.01–25 µL /mL for Trolox and the compounds. A solution of the tested compound/Trolox (50 µL) and DPPH solution in final concentration was placed in the 96-well microplate (Greiner Bio-One. Frickenhausen. Germany). The plate was immediately placed in a multifunctional microplate reader (Synergy H1. BioTek). Measurements of the absorbance were made at room temperature using a microplate reader. The wavelength was 517 nm. As a blank, 75 mM phosphate buffer (pH 7.4) was used instead of the tested compounds/Trolox. The DPPH stability during measurements was obtained by the addition of 75 mM phosphate buffer (pH 7.4) instead of the compounds. The plate was automatically shaken before each measurement for 5 s. All tests were performed in triplicate for each sample [61].

### 3.9. ORAC-FL

Oxygen Radical Absorbance Capacity-Fluorescein (ORAC-FL) is a method that uses oxygen for measuring the antioxidant activity of selected compounds and Trolox as standard. In this method were used fluorescein disodium salt (FL), 2,2′-azobis(2-methylpropionamidine) dihydrochloride (APPH), and 6-hydroxy-2,5,7,8-tetramethyl-chromane-2-carboxylic acid (Trolox) as a standard. These chemical reagents were purchased from Sigma-Aldrich (Steinheim, Germany). The assays of selected compounds and the standard were carried out in 75 mM phosphate buffer (pH 7.4) with a final reaction volume of 200 µL. All reagents were dissolved in 75 mM phosphate buffer (pH 7.4). The final concentrations were 1–13 µM for both Trolox and compounds. A solution of all the compounds (20 µL) and fluorescein (120 µL, 70 nM final concentration) were placed in the well of the black 96-well microplate (Greiner Bio-One, Frickenhausen, Germany). The mixtures of compounds and fluorescein were incubated in a dark place at 37 °C for 15 min. After incubation, 60 µL of APPH (12 mM, final concentration) was quickly added for an indication of the reaction. The plates were immediately put in a multifunctional microplate reader (Synergy H1, BioTek). The fluorescence from the top was measured kinetically at 70 s intervals for 140 min at 37 °C using a microplate reader. Shifts of fluorescence were measured at 485 nm excitation and 520 nm emission using fluorescent monochromators. Phosphate buffer (pH 7.4) was used as a blank assay in place of the tested compounds/Trolox. The fluorescein stability during measurements was checked by adding the same buffer in place of the tested compounds/Trolox and APPH. The plate was automatically shaken before each measurement for 10 s. All tests were performed in three independent runs for each sample [51].

### 3.10. Cell Culture and MTT Cytotoxicity Assay

Activities of the new compounds were tested against A549 cells obtained from human lung adenocarcinoma (non-small-cell cancer cells). A549 cells were obtained from the European Collection of Cell Cultures (ECACC, Salsburg, Loughborough, UK). The cells were cultured with Dulbecco’s Modified Eagle’s medium (DMEM) containing 10% FBS (fetal bovine serum, Merck), 100 U/mL penicillin (Merck), and 100 mg/mL streptomycin (Merck) at 37 °C with 5% CO_2_. DMSO (Merck) was used to prepare stock solutions for new compounds.

The MTT assay was used to evaluate anticancer activity of the new compounds. This test is based on the determination of cell viability. Viable cells convert water-soluble MTT (3-(4,5-dimethylthiazol-2-yl)-2,5-diphenyltetrazolium bromide) (Merck) compound to blue–violet insoluble formazan product. A549 were seeded at a concentration of 104 cells/well in 96-well plates. After 24 h, the medium was removed, and cells were treated with new compounds in 10, 50, 100, 200, 400, and 600 μM concentration for the next 24 h. For each well, the medium was removed, and MTT reagent (0.5 mg/mL) was added, and then cells were kept in the dark for 2 h at 37 °C. Next, the MTT solution was removed and replaced with 100 μL of DMSO (Merck), and the absorbance was measured at 570 nm (Synergy H1, BioTek, Winooski, VT, USA) after 10 min.

## 4. Conclusions

A series of new thiosemicarbazone derivatives (L–L5) with various substituents in the phenyl ring were synthesized. The structure of the compounds was confirmed using 1H NMR, 13C NMR, FTIR spectroscopy, and high-resolution mass spectrometry. Antibacterial activity studies showed that compound L1 exhibits the strongest antibacterial effect, especially against Bacillus cereus ŁOCK 0807 (MIC 10 mg/L). Compounds L and L2 also demonstrated significant antibacterial activity against selected strains. Molecular docking suggests that all studied compounds can effectively bind to selected molecular targets—topoisomerase II and transcriptional regulator PrfA—indicating their anticancer and antibacterial potential. Analysis of physicochemical properties (ADME) confirmed that all compounds meet Veber’s and Lipinski’s rules, suggesting their potential oral bioavailability. Antioxidant activity studies using ABTS, DPPH, and ORAC-FL assays revealed that compounds L, L1, and L2 possess the strongest antioxidant properties, comparable to or better than the standard—Trolox. The MTT cytotoxicity test on the A549 cell line indicated that introducing L1 and L2 substituents does not significantly affect cell survival compared to the parent compound L. At the same time, the L4 derivative shows the strongest anticancer potential. We analyzed the structure of the compounds as a structure–activity relationship. Thus, it should be noted that the new thiosemicarbazide derivatives have a structure similar to ambazone. Ambazone has an amino thiourea group, which is responsible for antimicrobial activity, among other things. It shows particular activity against Gram-positive bacteria. On the other hand, free electron pairs in nitrogen atoms affect oxidative stress created by inflammation. The presence of bacteria in the body causes inflammation, which is manifested by changes in oxidative stress on cells in the inflamed area. Referring to the aromatic ring in the structure of the compounds we obtained, it should be noted that a significant proportion of antimicrobial substances are simple structures containing a five- or six-membered aromatic ring. The above-mentioned elements of the structure and the results of the analyses indicate that the designed compounds fall into the trend of antimicrobial drugs.

## Figures and Tables

**Table 1:** The route of synthesis of compounds **L–L5**.

Compound	R1	R2
L	H	2-pyridine
L1	2-Cl	2-pyridine
L2	3-Cl	2-pyridine
L3	4-Cl	2-pyridine
L4	2,4-diCl	2-pyridine
L5	2,6-diCl	2-pyridine

**Table 2:** High-resolution mass spectrometry results compared to the theoretical values.

Compound	[M+H]^+^_theor._	[M+H]^+^_meas._	Delta [ppm]
L	257.0855	257.0847	−3.4
L1	291.0466	291.0458	−2.7
L2	291.0466	291.0458	−2.7
L3	291.0466	291.0456	−3.3
L4	325.0076	325.0065	−3.1
L5	325.0076	325.0069	−2.1

**Table 3:** Docking scores of the studied compounds and original inhibitors calculated as a total ChemPLP model fitness value.

Compound	TopoII	PrfA
L	76.72	64.35
L1	72.70	58.56
L2	72.81	58.86
L3	70.15	55.80
L4	73.10	54.40
L5	71.47	56.60
Original inhibitor	110.45 *	73.18 **

* PDB ID: EVP (etoposide); ** PDB ID: QQH

**Table 4:** The minimum inhibitory concentration (MIC) of the investigated compounds inhibited the growth of the tested microorganisms.

Chemicals	L	L1	L2	L3	L4	L5	Ref. (Van/Cip)
Microorganism	MIC [mg/L]						
*Bacillus cereus* ŁOCK 0807	1000	10	>1000	100	50	>1000	1
*Bacillus subtilis* ATCC 6633	200	100	50	200	1000	>1000	1
*Enterococcus faecalis* ATCC 29212	>1000	>1000	>1000	>1000	>1000	>1000	2
*Staphylococcus aureus* ATCC 25923	1000	1000	50	1000	>1000	1000	1
*Staphylococcus aureus* ATCC 6538	1000	100	500	1000	100	1000	1
*Staphylococcus epidermidis* ATCC 12228	200	100	>1000	>1000	>1000	>1000	1
*Listeria monocytogenes* ATCC 19115	200	100	500	>1000	1000	1000	2
*Escherichia coli* ATCC 10530	1000	>1000	1000	>1000	>1000	1000	1
*Salmonella typhimurium* ATCC 14028	>1000	>1000	>1000	>1000	>1000	1000	1

Reference antibacterial agents (Ref.): Vancomycin (Van) for Gram-positive bacteria/ciprofloxacin (Cip) for Gram-negative bacteria.

**Table 5:** ABTS results for assessing the total antioxidant capacity of compounds **L–L5**.

Compound	IC50 (mg/mL)	SD (+/−)
L	0.010	0.001
L1	0.017	0.004
L2	0.012	0.003
L3	0.015	0.002
L4	0.107	0.053
L5	0.017	0.052
Trolox (STD) *	0.015	0.002

* Trolox equivalent.

**Table 6:** DPPH results for assessing the total antioxidant capacity of compounds **L–L5**.

Compound	IC50 (mg/mL)	SD (+/−)
L	0.403	0.058
L1	0.088	0.008
L2	0.025	0.001
L3	-	-
L4	0.160	0.003
L5	0.141	0.026
Trolox (STD) *	0.013	0.002

* Trolox equivalent.

**Table 7:** ORAC-FL results for assessing the total antioxidant capacity of compounds **L–L5**.

Compound	IC50 (mg/mL)	SD (+/−)
L	0.028	0.001
L1	0.010	0.001
L2	0.021	0.001
L3	0.020	0.002
L4	0.025	0.002
L5	0.033	0.002
Trolox (STD) *	1.000	0.000

* Trolox equivalent.

**Table 8:** Cell screening survival values. All values are presented as the means ± standard deviation (SD).

Compound	IC50 (µM)	SD (+/−)
L	510.61	11.53
L1	479.96	13.12
L2	524.89	10.25
L3	609.18	15.21
L4	193.44	8.20
L5	858.93	36.21
Cisplatin	841.25	12.02
Doxorubicin	194.65	21.01

## Data Availability

Data is contained within the article or Supplementary Material.

## Supplementary Materials

The following supporting information can be downloaded at: https://www.mdpi.com/article/10.3390/molecules30010129/s1, Figure S1. a. 1H NMR spectrum of the L compound. b. ^13^C NMR spectrum of the L compound. c. ^1^H NMR spectrum of the L1 compound. d. 13C NMR spectrum of the L1 compound. e. ^1^H NMR spectrum of the L2 compound. f. 13C NMR spectrum of the L2 compound. g. ^1^H NMR spectrum of the L3 compound. h. ^13^C NMR spectrum of the L3 compound. i. ^1^H NMR spectrum of the L4 compound. j. ^13^C NMR spectrum of the L4 compound. k. ^1^H NMR spectrum of the L5 compound. l. ^13^C NMR spectrum of the L5 compound. Figure S2. a. ESI mass spectrum of the L compound in positive ion mode. Ion at *m*/*z* 257.0847 corresponds to the protonated L molecule. b. ESI mass spectrum of the L1 compound in positive ion mode. Ion at *m*/*z* 291.0458 corresponds to the protonated L1 molecule. c. ESI mass spectrum of the L2 compound in positive ion mode. Ion at *m*/*z* 291.0458 corresponds to the protonated L2 molecule. d. ESI mass spectrum of the L3 compound in positive ion mode. Ion at *m*/*z* 291.0456 corresponds to the protonated L3 molecule. e. ESI mass spectrum of the L4 compound in positive ion mode. Ion at *m*/*z* 325.0065 corresponds to the protonated L4 molecule. f. ESI mass spectrum of the L5 compound in positive ion mode. Ion at *m*/*z* 325.0069 corresponds to the protonated L5 molecule. Figure S3a–f. FTIR spectra of compounds **L–L5**.
