# Supplementary material for: Thiosemicarbazone-Based Compounds: A Promising Scaffold for Developing Antibacterial, Antioxidant, and Anticancer Therapeutics
Source: Molecules. 2024 Dec 31;30(1):129. doi: 10.3390/molecules30010129 (PMC11721278; doi:10.3390/molecules30010129)
Supplement: Supplementary file 1 [file molecules-30-00129-s001.zip › molecules-3350156-supplementary.pdf]

Supplementary Materials

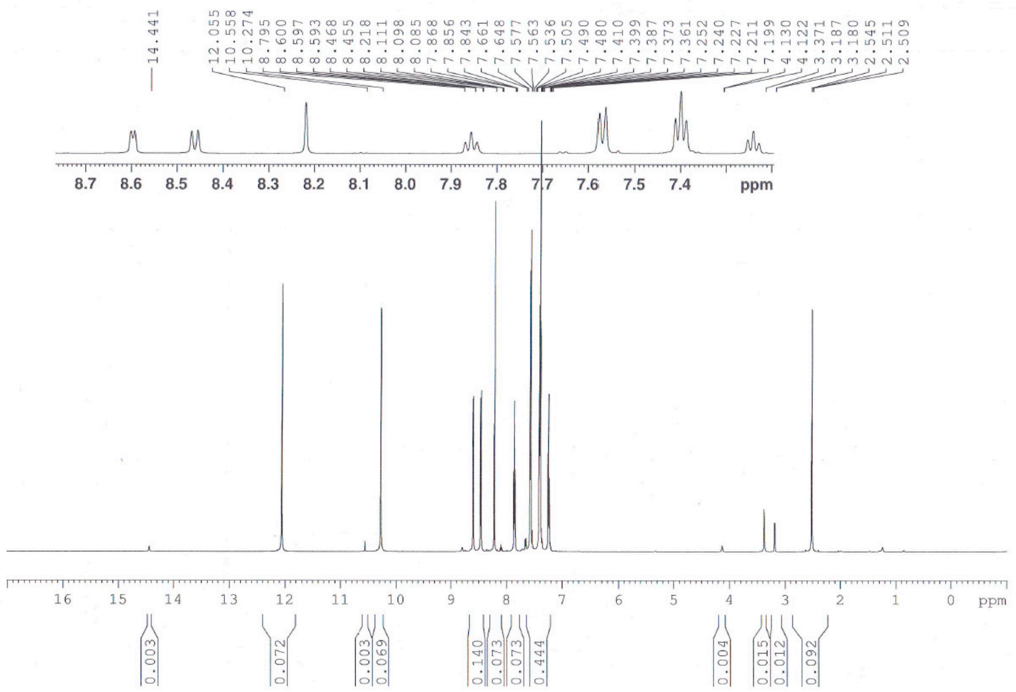

Figure S1. a. ^{1}H NMR spectrum of the **L** compound.

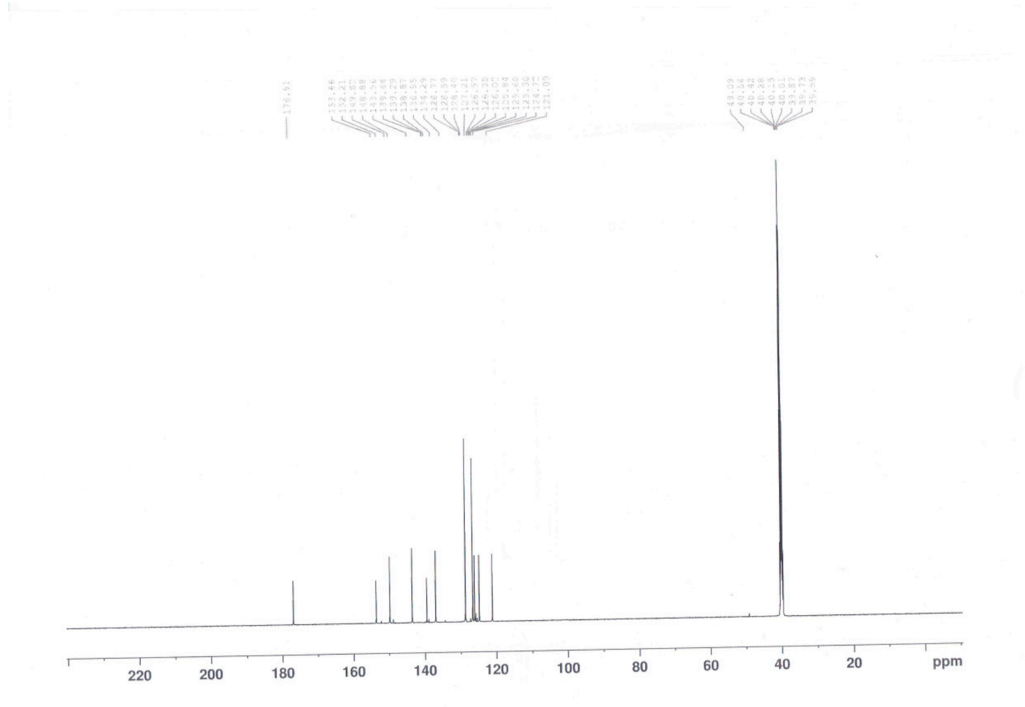

Figure S1. b. ^{13}C NMR spectrum of the **L** compound.

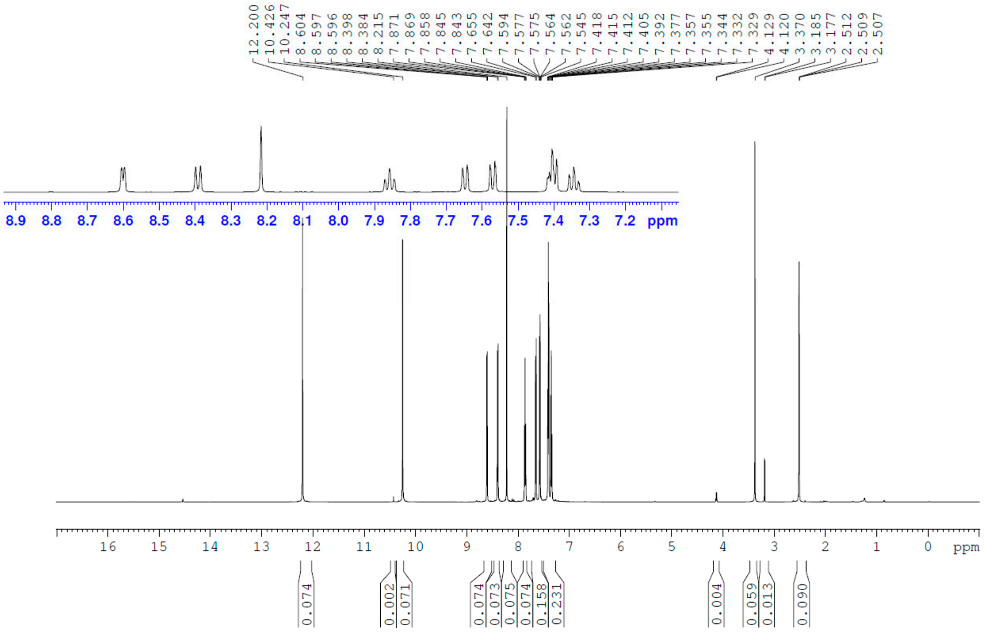

Figure S1. c. ¹H NMR spectrum of the **L1** compound.

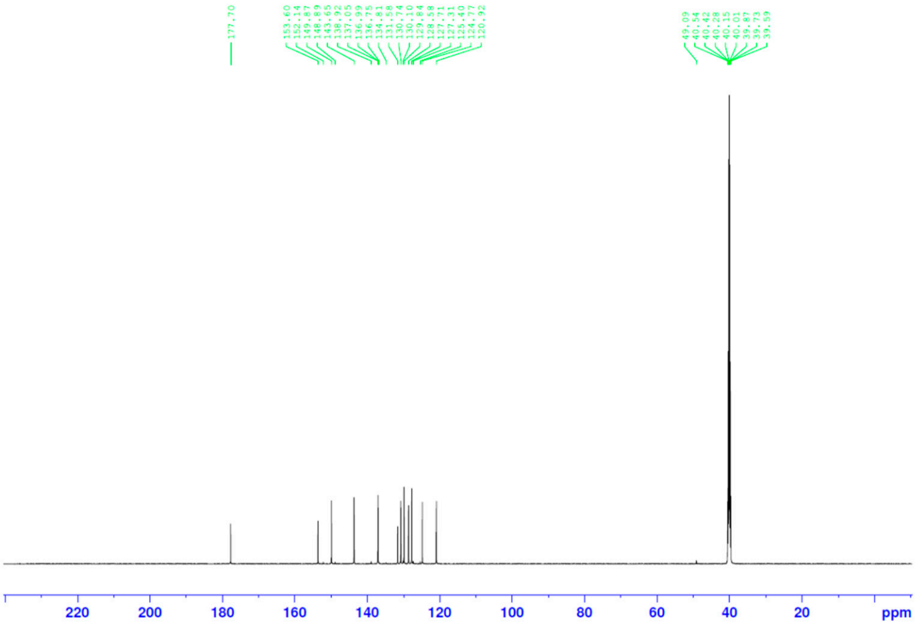

Figure S1. d. ¹³C NMR spectrum of the **L1** compound.

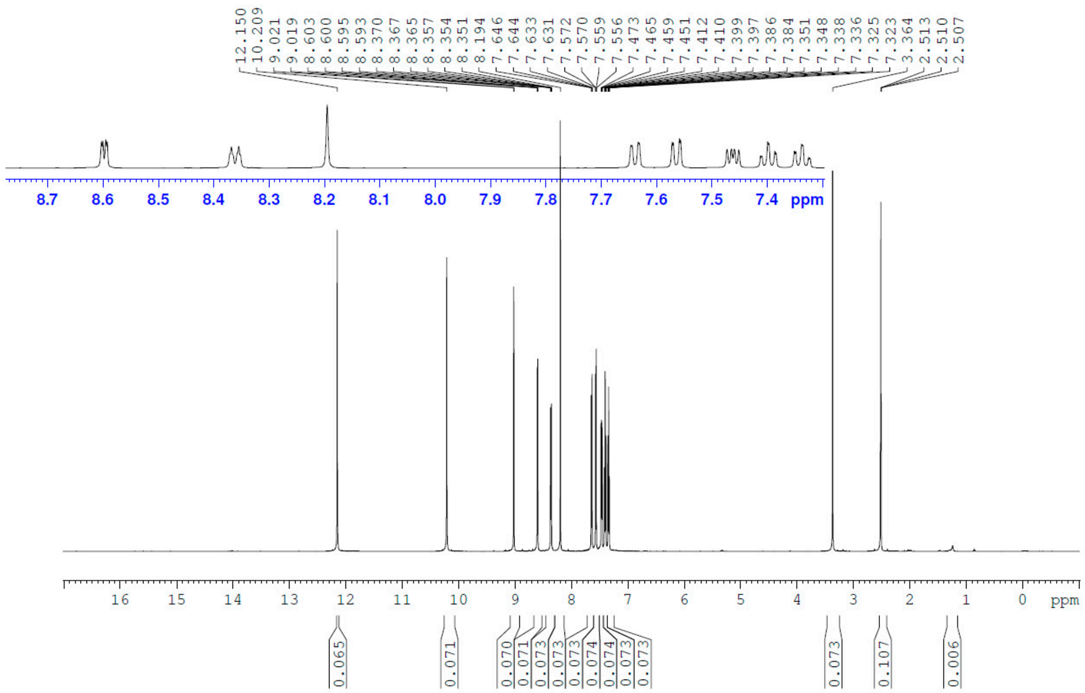

Figure S1. e. ^{1}H NMR spectrum of the **L2** compound.

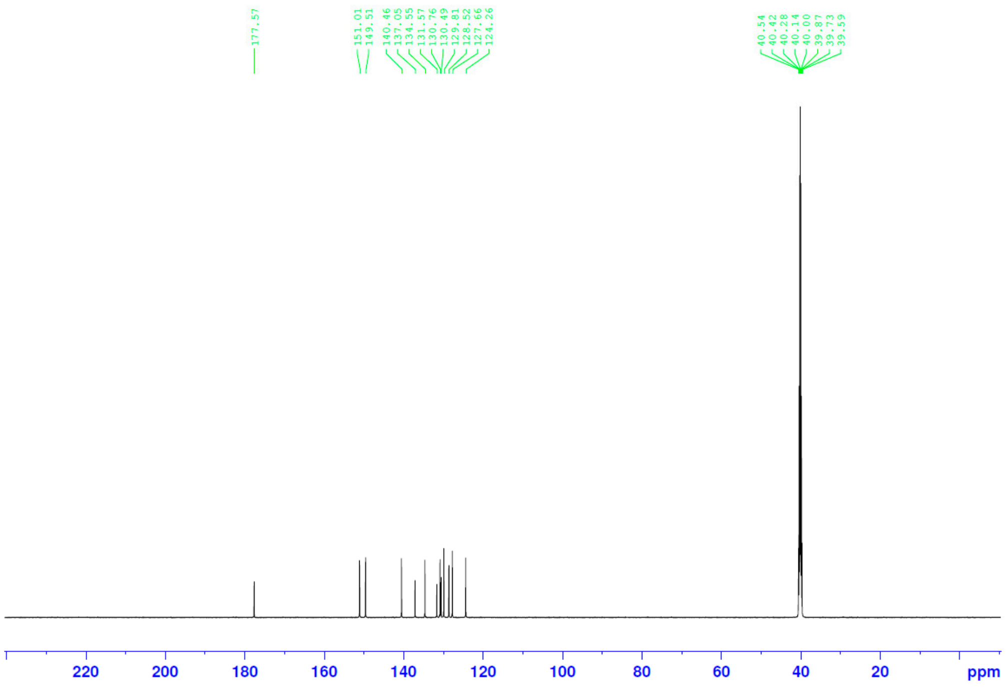

Figure S1. f. ^{13}C NMR spectrum of the **L2** compound.

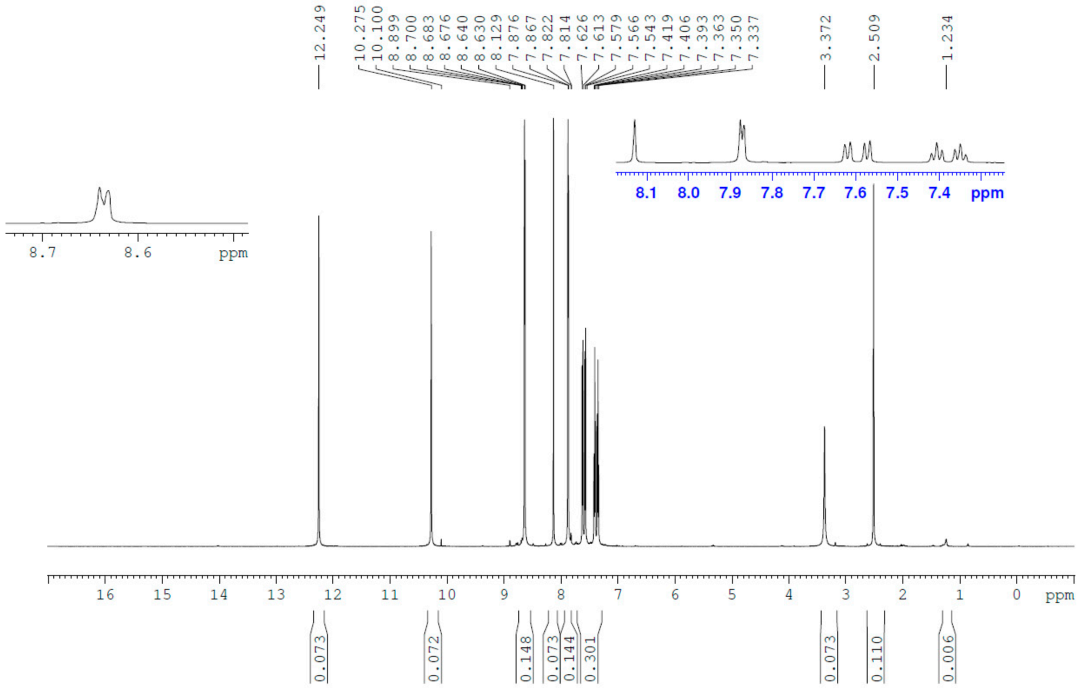

Figure S1. g. ^{1}H NMR spectrum of the **L3** compound.

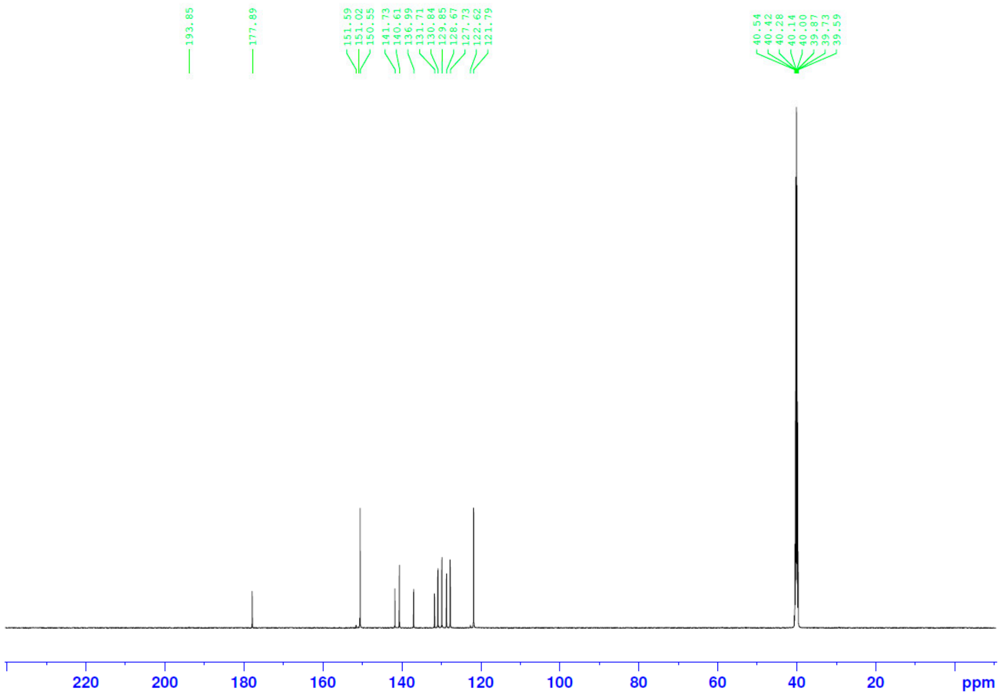

Figure S1. h. ^{13}C NMR spectrum of the **L3** compound.

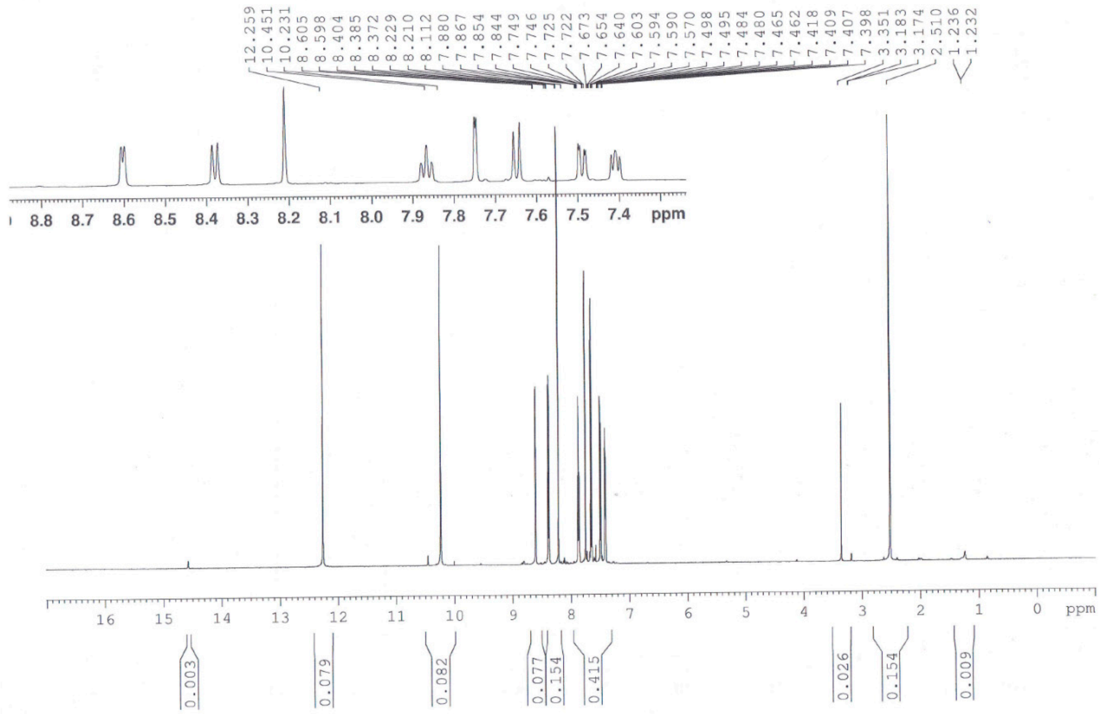

Figure S1. i. ^{1}H NMR spectrum of the **L4** compound.

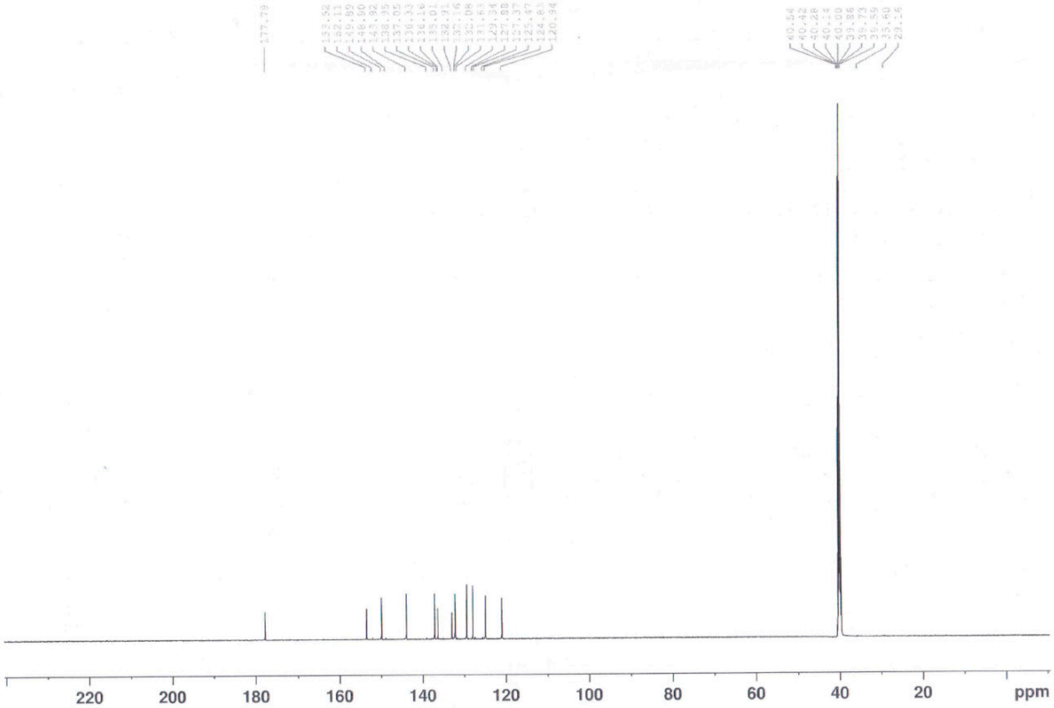

Figure S1. j. ^{13}C NMR spectrum of the **L4** compound.

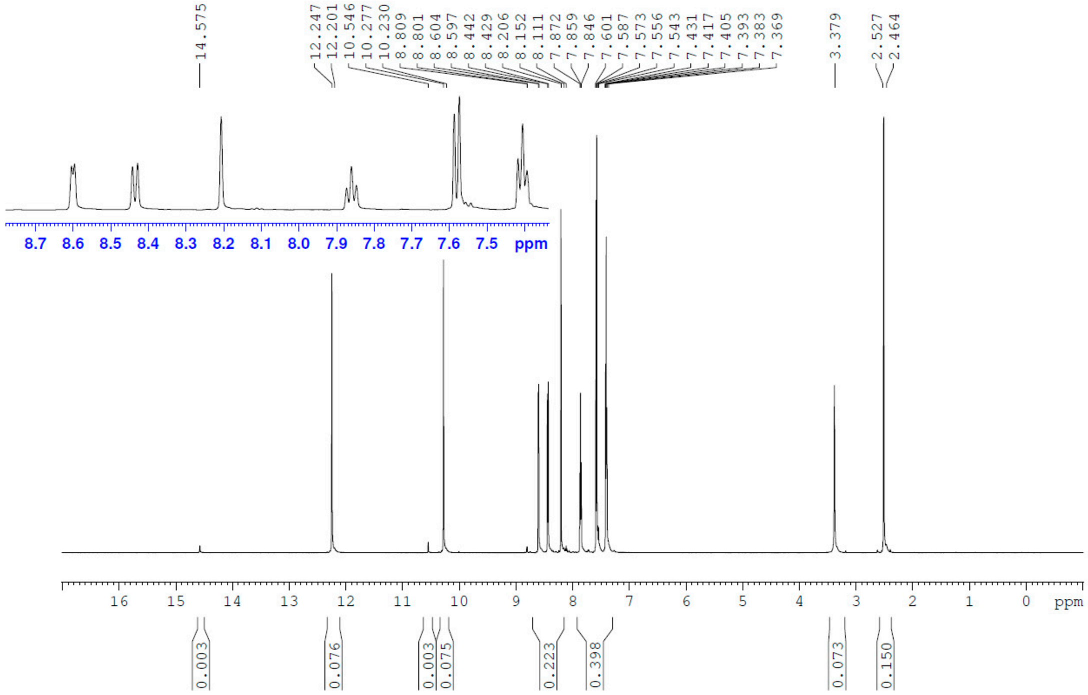

Figure S1. k. ^{1}H NMR spectrum of the **L5** compound.

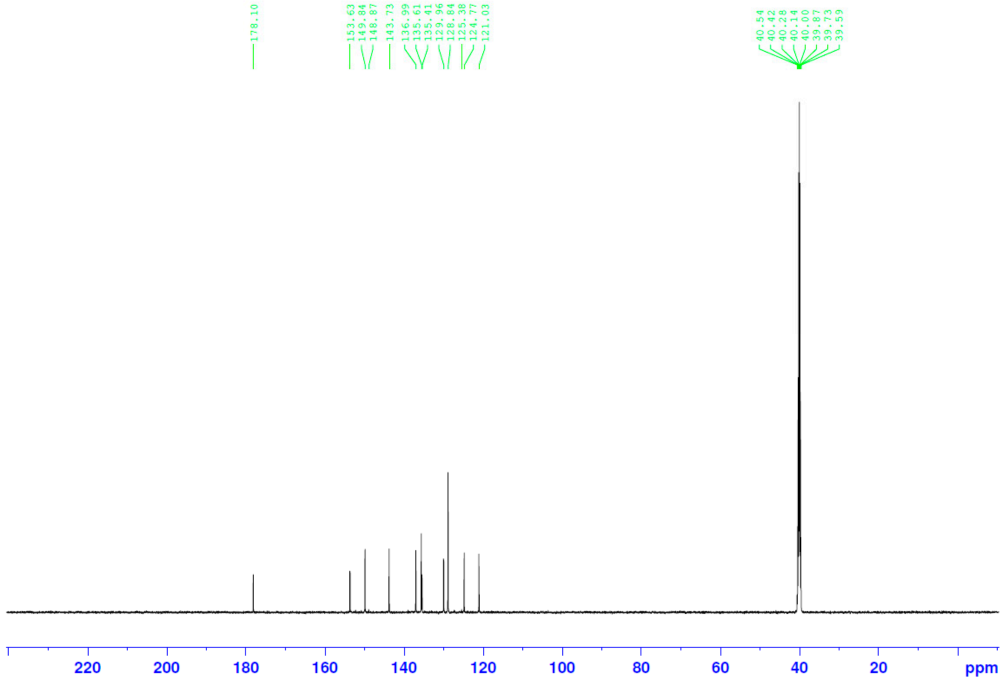

Figure S1. l. ^{13}C NMR spectrum of the **L5** compound.

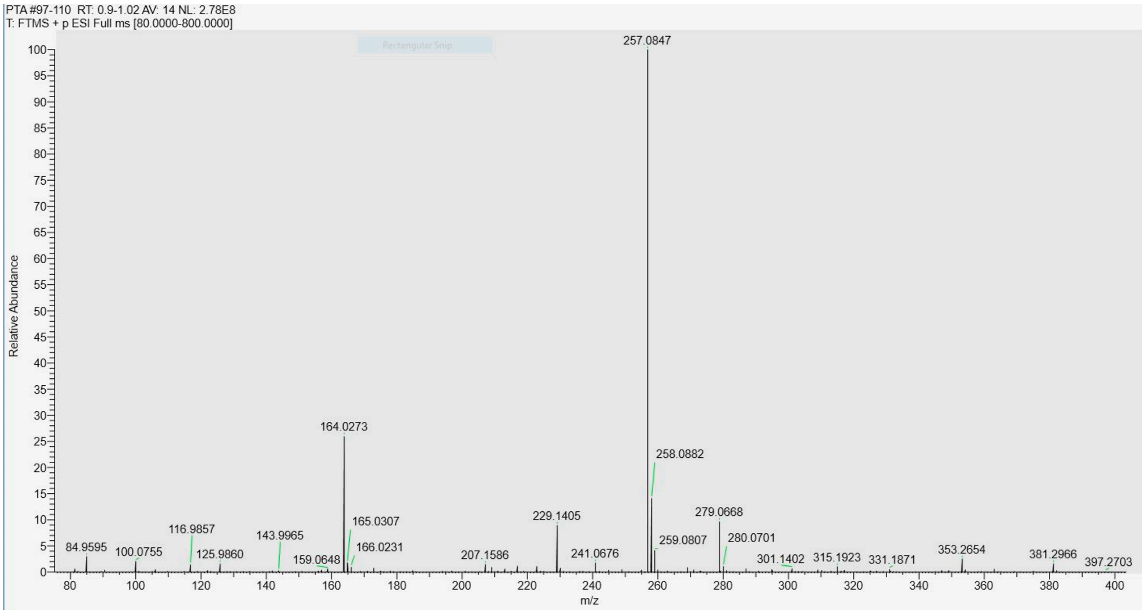

Figure S2. a. ESI mass spectrum of the **L** compound in positive ion mode. Ion at m/z 257.0847 corresponds to the protonated **L** molecule.

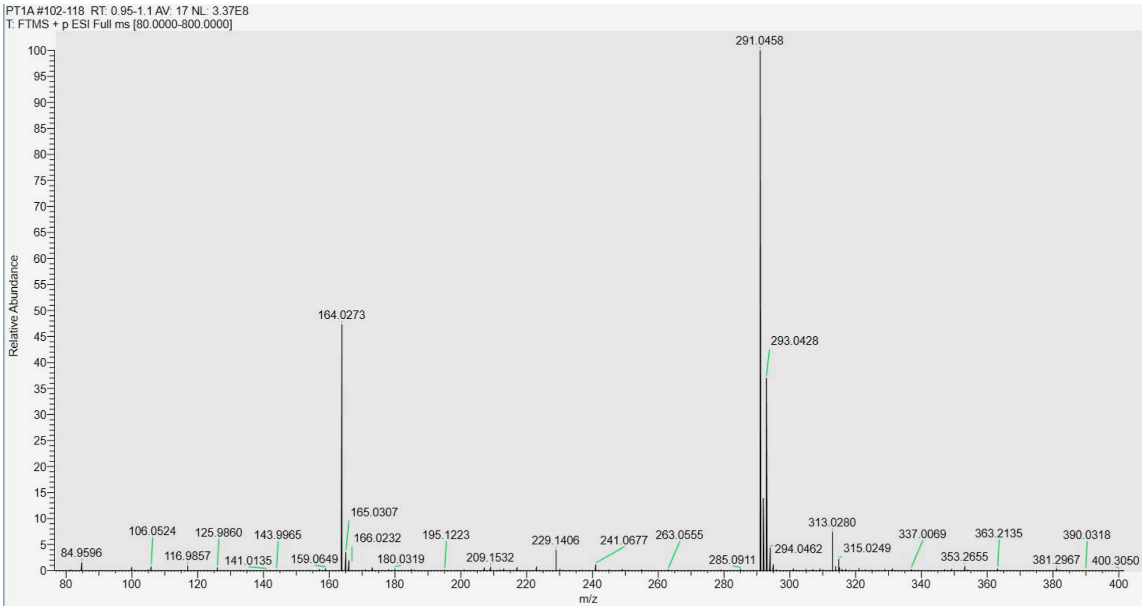

Figure S2. b. ESI mass spectrum of the **L1** compound in positive ion mode. Ion at m/z 291.0458 corresponds to the protonated **L1** molecule.

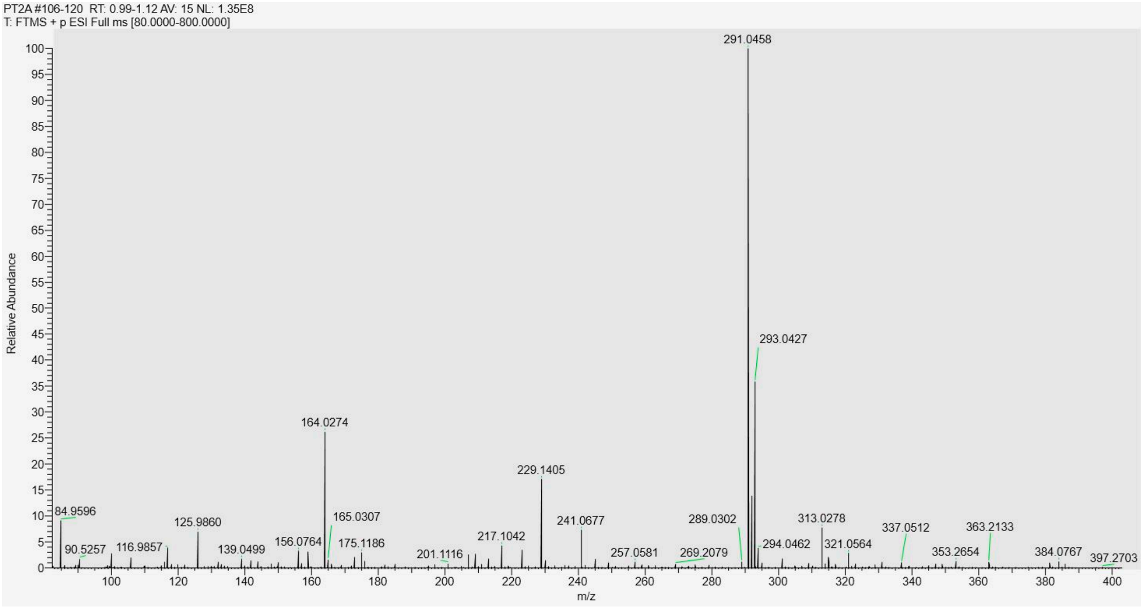

Figure S2. c. ESI mass spectrum of the **L2** compound in positive ion mode. Ion at m/z 291.0458 corresponds to the protonated **L2** molecule.

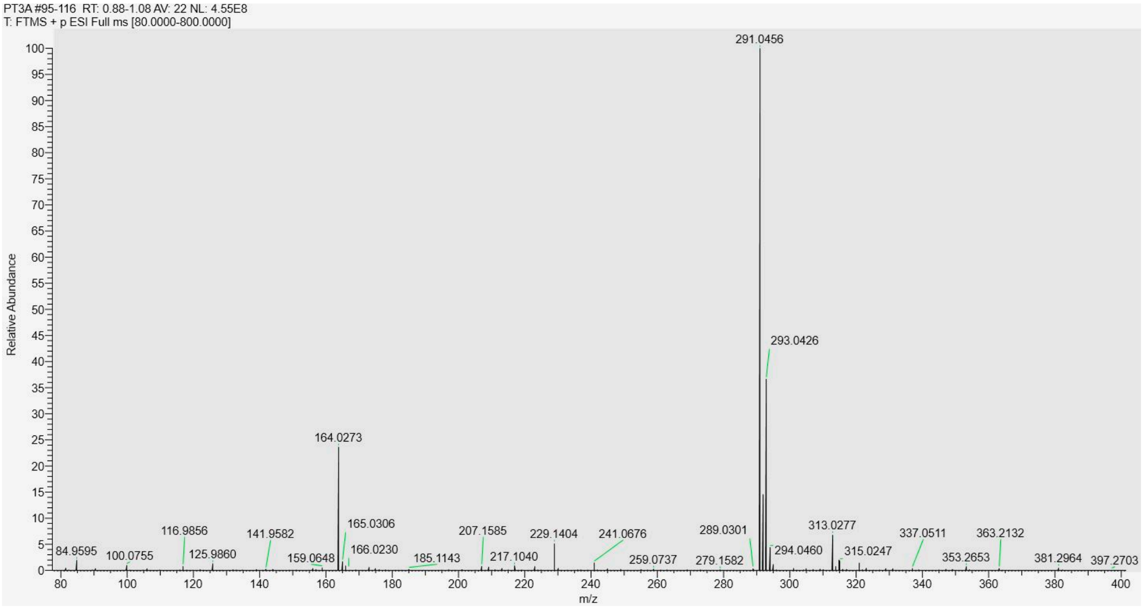

Figure S2. d. ESI mass spectrum of the **L3** compound in positive ion mode. Ion at m/z 291.0456 corresponds to the protonated **L3** molecule.

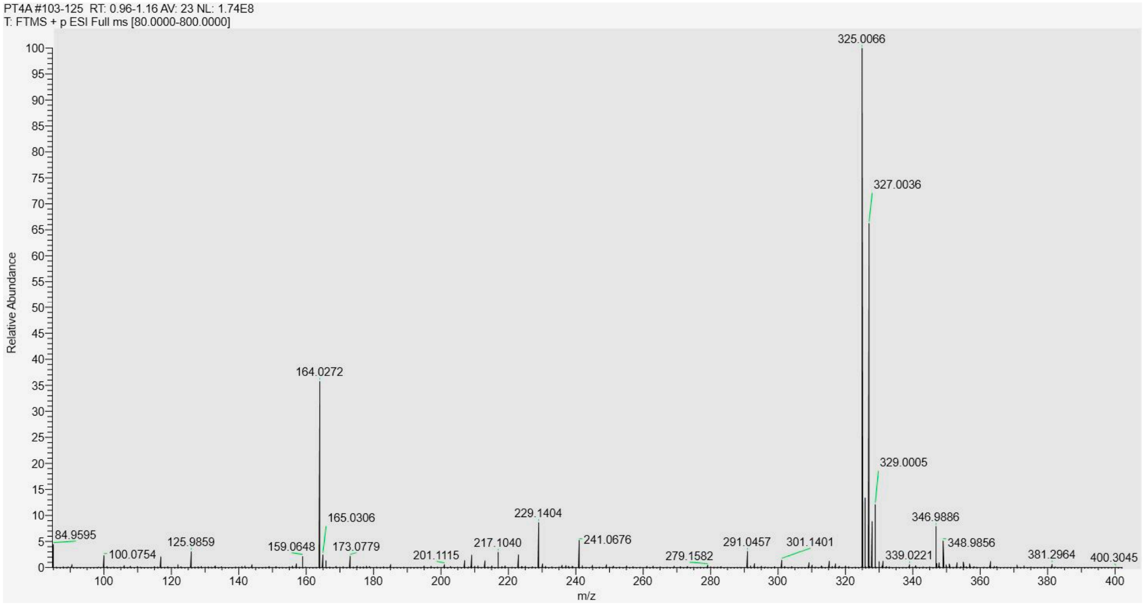

Figure S2. e. ESI mass spectrum of the **L4** compound in positive ion mode. Ion at m/z 325.0065 corresponds to the protonated **L4** molecule.

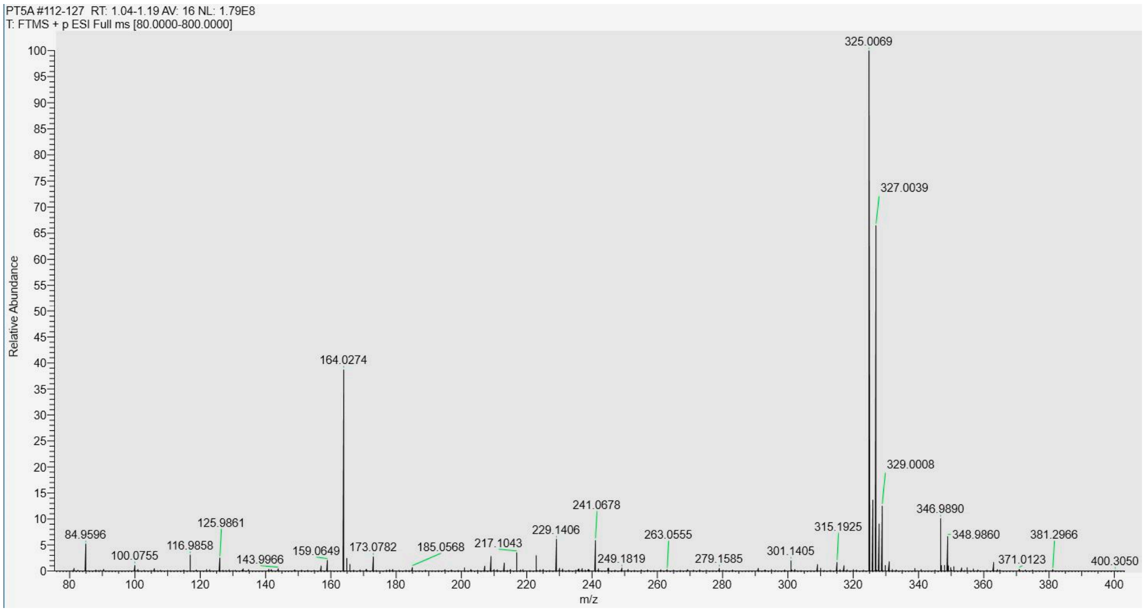

Figure S2. f. ESI mass spectrum of the **L5** compound in positive ion mode. Ion at m/z 325.0069 corresponds to the protonated **L5** molecule.

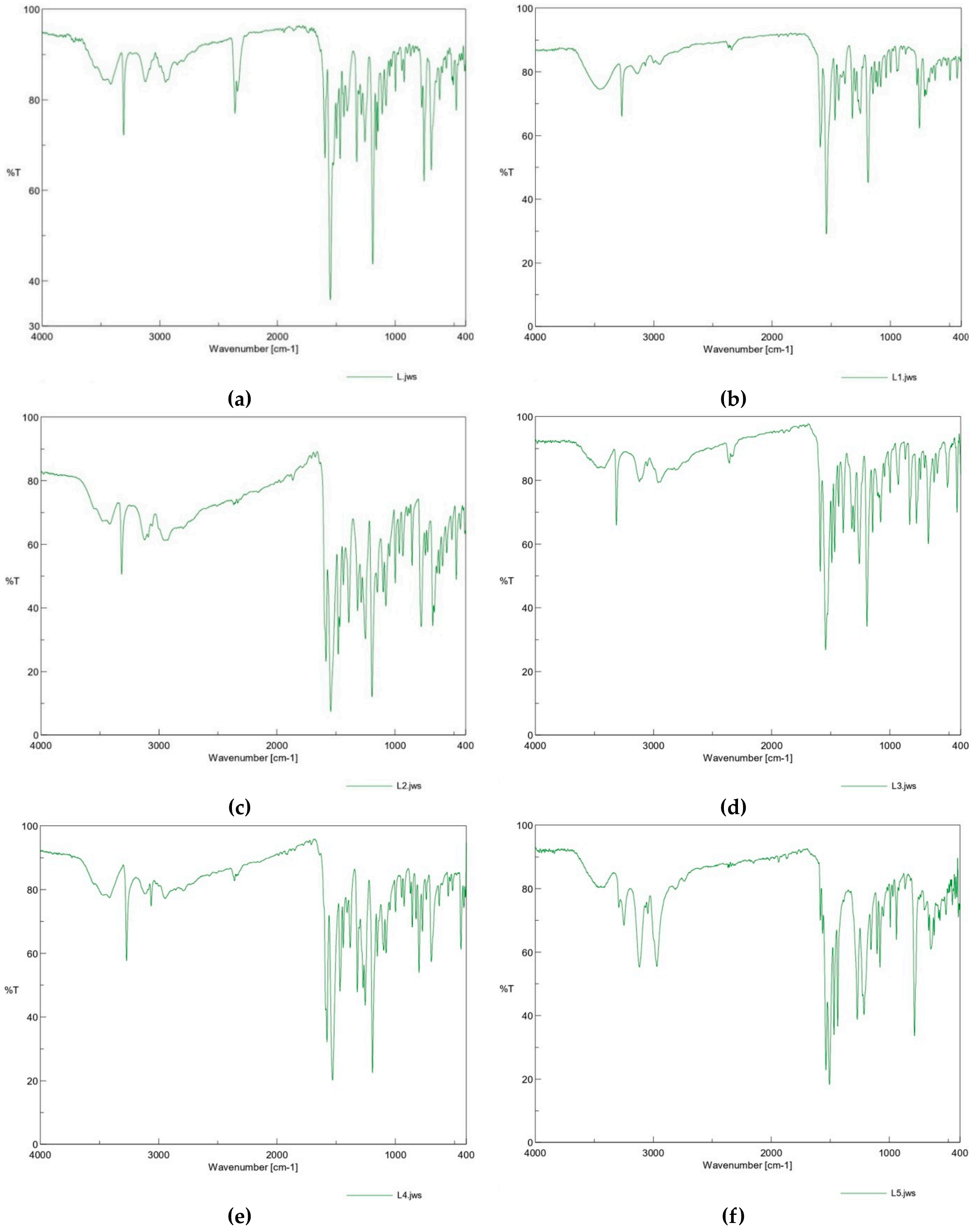

Figure S3. a–f. FTIR spectra of compounds **L-L5**.
